# Antibacterial activity of *Moringa* leaf extracts against Gram-negative bacteria from Wadi Ad-Dawasir, Saudi Arabia

**DOI:** 10.3389/fmicb.2025.1568105

**Published:** 2026-01-12

**Authors:** Maha S. I. Wizrah, Nourah D. Aldwsari, Zeinab A. Yahia

**Affiliations:** Department of Biology, College of Sciences and Humanities in Al-Kharj, Prince Sattam Bin Abdulaziz University, Al-Kharj, Saudi Arabia

**Keywords:** antimicrobial resistance, Gram-negative bacteria, *Moringa oleifera*, *Moringa peregrina*, phytochemicals, Wadi Ad-Dawasir

## Abstract

**Background:**

*Moringa oleifera* and *Moringa peregrina* have been extensively studied for their medicinal properties, particularly due to their rich content of bioactive phytocompounds. While their antibacterial effects on Gram-positive bacteria are well documented, there is a paucity of comparative studies on their efficacy against Gram-negative multidrug-resistant (MDR) strains found in arid regions.

**Methods:**

Leaf extracts of *M. oleifera* and *M. peregrina,* collected from Wadi Ad-Dawasir in Saudi Arabia, were prepared using ethanol, hot aqueous, and cold aqueous extraction methods. Their antibacterial properties were tested against *Escherichia coli*, *Pseudomonas aeruginosa*, *Shigella sonnei*, and *Shigella shiga* using the agar well diffusion method, minimum inhibitory concentration (MIC) assays (50–200 μg/mL), and scanning electron microscopy (SEM). Phytochemical analysis was conducted using gas chromatography–mass spectrometry (GC–MS) and standard qualitative methods. Amoxicillin–clavulanic acid and tetracycline were used as reference antibiotics.

**Results:**

All extracts showed antibacterial activity, with ethanol extracts producing the largest inhibition zones. *M. peregrina* was particularly effective, especially against *Sh. sonnei*, with an inhibition zone of 24.66 ± 0.17 mm, which surpassed that of amoxicillin–clavulanic acid (18.25 ± 1.69 mm). The MIC values were generally 50 μg/mL for both species, except for the cold extract of *M. oleifera* against *P. aeruginosa*, which had an MIC of 100 μg/mL. GC–MS analysis identified 31 compounds in *M. peregrina*, with stigmasterol and *β*-sitosterol being the most abundant. In contrast, *M. oleifera* was characterized by a profile rich in fatty acids. The SEM analysis revealed membrane damage in *Sh. sonnei* treated with the extracts, which included surface collapse, perforation, and cytoplasmic leakage.

**Conclusion:**

Both *Moringa species* demonstrated significant antibacterial activity against clinically important Gram-negative strains, with *M. peregrina* occasionally outperforming standard antibiotics. The presence of sterols in the phytochemical profile likely enhances their bactericidal action by targeting bacterial membranes. These results suggest that *Moringa* could serve as a viable phytotherapeutic option for addressing the growing challenge of Gram-negative bacterial resistance.

## 1 Introduction

Bacterial infections are increasingly becoming life-threatening, with projections indicating that they may pose a greater risk to human health than cancer and become more challenging to treat (Murray et al., 2022). The primary factor contributing to this threat is the development of resistance by bacterial pathogens to antibacterial agents. Antibiotic resistance is associated with elevated rates of morbidity and mortality, thereby representing a significant threat to public health. The ineffectiveness of traditional antibiotics against infections caused by both Gram-positive and Gram-negative bacteria has been exacerbated by multidrug resistance, complicating treatment efforts (Velez and Sloand, 2016; Sharifi-Rad et al., 2025). Addressing this issue necessitates the discovery of new antibacterial agents to replace those rendered ineffective by resistance (Medina et al., 2016; Ziebuhr et al., 1999; Andersson, 2003). Furthermore, the use of antibiotics in agriculture and animal husbandry significantly contributes to the emergence and spread of antimicrobial resistance. This problem is further aggravated when antibiotics are used for non-therapeutic purposes, such as promoting growth in livestock—a practice prohibited in many regions, including the European Union since 2006, due to their established role in accelerating resistance. Resistant bacteria that originate from animal production systems can be transmitted to humans through the food chain, thereby posing a substantial public health concern (Manyi-Loh et al., 2018). However, the development of new antibacterial agents alone is insufficient, as the misuse and overuse of antibiotics in human medicine can also lead to drug resistance and recurrent infections (Rhen et al., 2003). Historically, traditional medicine has utilized plants and their extracts to treat bacterial and fungal infections. Consequently, the pharmaceutical industry is increasingly focused on developing new drugs derived from natural resources. Numerous studies have investigated the antibacterial efficacy of traditional plants against microorganisms (Sharifi Rad et al., 2014; Bugno et al., 2007). These plants contain various secondary metabolites such as phenolic compounds and alkaloids that exhibit antimicrobial properties (Sharifi-Rad et al., 2022; Benmohamed et al., 2023). Alternative treatments are gaining popularity in developing countries, partly due to the World Health Organization guidelines. This trend has prompted experimental and clinical research that underscores the significance of plants in medicine (Vijaya and Ananthan, 1997; Dilhuydy, 2003).

Plants are recognized as safe, cost-effective, accessible, and potent antimicrobial agents for treating a wide range of diseases (Sharif and Banik, 2006; Doughari et al., 2007). Although 28,187 plant species have been documented for medicinal use, only 4,478 are cited in regulatory literature (Willis, 2017). Among these, *Moringa*, a genus in the Moringaceae family, is notable for its significant contributions to traditional medicine, pharmaceuticals, and the identification of bioactive components. This genus comprises 13 species, including *M. oleifera* and *M. peregrina* (Quattrocchi, 2012; Fahey et al., 2018). *M. oleifera*, in particular, is of considerable economic and medicinal value. It is often referred to as the “miracle tree,” “drumstick tree,” or “the tree of life” (Peter, 1979; Banerji et al., 2003; Morton, 1991; Sengupta and Gupta, 1970)and is native to India and the sub-Himalayan regions of Northern India (Sharma et al., 2011; Pandey et al., 2012). In addition, *M. oleifera* is cultivated and distributed across tropical regions of Africa, Arabia, Central America, North America, South America, the Philippines, Cambodia, Bangladesh, Pakistan, and Sri Lanka (Fahey, 2005). This plant has various medicinal properties, including cholesterol-lowering, anticancer, anti-inflammatory, antioxidant, and antidiabetic effects (Qixia et al., 2020; Xu et al., 2024).

The various components of *M. oleifera* are highly nutritious, with the leaves being particularly rich in proteins, vitamins, and minerals (Avilés-Gaxiola et al., 2021; Cao et al., 2023). Extensive research on both fresh and dried leaves of *M. oleifera* has revealed that the dried leaves contain higher concentrations of vitamin B, fiber, nutrients, carbohydrates, protein, copper, potassium, magnesium, calcium, phosphorus, and iron than fresh leaves. Conversely, fresh leaves exhibit elevated levels of vitamins C and E (Brilhante et al., 2017; Abe and Ohtani, 2013; Trigo et al., 2020). It has been established that fresh leaves possess higher amounts of vitamins A and C than carrots and oranges (Fahey, 2005). *M. peregrina*, the second most significant species within the Moringaceae family, is primarily found in the Arabian Peninsula and the region surrounding the Red Sea (Boulos, 2000). It holds potential as a functional food component (Hernandez-Aguilar et al., 2021). The seeds of *M. peregrina* are rich in oil, minerals, protein, and essential amino acids (Al-Dabbas et al., 2010; El-Hak et al., 2018). Previous studies on the *Moringa* genus have predominantly focused on *M. oleifera*, particularly its antioxidant activities, while the antibacterial properties of *M. peregrina* have received limited attention. A notable research gap exists in the comparative analysis of species from arid regions such as Wadi Ad-Dawasir, particularly regarding their efficacy against Gram-negative bacteria in light of the rising issue of antibiotic resistance. This study presents a comparative evaluation of the antibacterial efficacy of *M. oleifera* and *M. peregrina* leaf extracts against four clinically significant Gram-negative strains—*Sh. sonnei*, *Sh. Shiga, E. coli*, and *P. aeruginosa*—sourced from retrospective certified origins. Phytochemical profiling was conducted using gas chromatography–mass spectrometry (GC–MS) analysis and qualitative tests to correlate chemical signatures with observed inhibitory patterns. The objectives of this research are to assess the antibacterial activity of ethanol, hot aqueous, and cold aqueous extracts of *M. oleifera* and *M. peregrina* against the four Gram-negative bacterial species, to determine and compare the inhibition zones and MIC parameters with those of standard antibiotics, to characterize the phytochemical constituents that contribute to the observed antibacterial effects, and to visualize extract-induced bacterial ultrastructural changes using scanning electron microscopy (SEM).

## 2 Materials and methods

### 2.1 Plant sample collection and authentication

**Figure 1:**
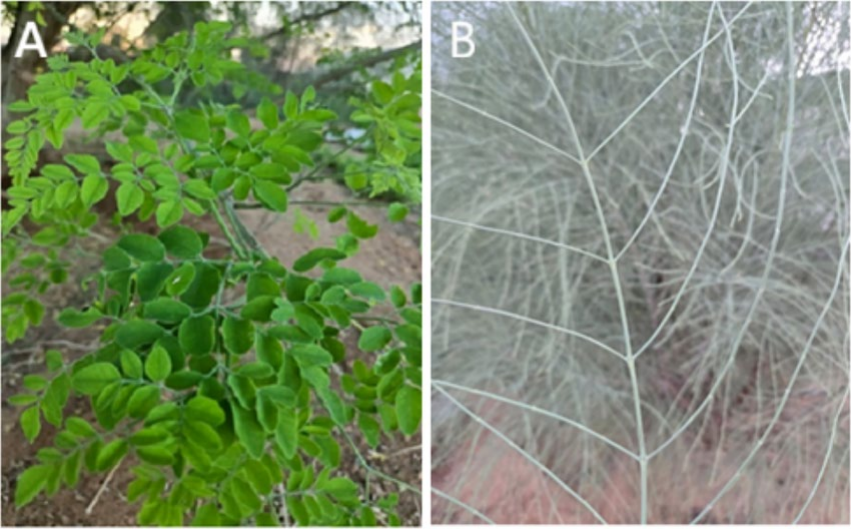
**(A)** *Moringa oleifera* leaves, characterized by bright green, pinnate leaflets. **(B)** *Moringa peregrina* leaves, noted for their slender, pale, thread-like appearance.

In April 2024, fresh leaves of *M. oleifera* and *M. peregrina* were collected from Wadi Ad-Dawasir, Saudi Arabia. A botanist at Prince Sattam Bin Abdulaziz University confirmed their identification. The collection process complied with the plant-handling guidelines set forth by the World Health Organization, which included shade drying the leaves at 25–30 °C for 2 weeks until a stable dry weight was achieved. The leaf samples were then stored at 4 °C until they were ready for extraction. Figures 1A,B illustrate the representative leaves of *M. oleifera* and *M. peregrina* used in this study.

### 2.2 Bacterial strains and biosafety compliance

The study utilized four archived Gram-negative bacterial isolates—*E. coli* MTCC 739, *P. aeruginosa* MTCC 2453, *Sh. sonnei* BMLRU1015, and *Sh. Shiga* BMLRU1013—obtained with certified documentation from the Animal Reproduction Research Institute in Egypt. These isolates were transported following the WHO’s triple-layer containment protocol at 4–8 °C, ensuring the integrity of the samples. As the study did not involve direct human or animal sampling, ethical approval was not necessary. The research exclusively employed archived bacterial isolates, with no direct patient sampling, thus exempting it from Institutional Review Board (IRB) review according to institutional and national guidelines.

### 2.3 Preparation of the extracts

#### 2.3.1 Preparation of the hot water extract

To prepare the hot aqueous extract, 50 g of leaves was boiled at 100 °C in 500 mL of distilled water (1:10 w/v) for 30 min, followed by a 24-h steeping period. The resulting filtrates were lyophilized and subsequently stored at 4 °C.

#### 2.3.2 Preparation of the cold water extract

For the cold aqueous extract, 50 g of leaves was immersed in cold sterile deionized water (4 °C) for 24 h. The mixture was then filtered, freeze-dried, and stored at 4 °C (Bhattacharya and Chandra, 2014).

#### 2.3.3 Preparation of the ethanol extract

The ethanol extract was obtained by performing Soxhlet extraction using 100 g of dried leaves and 95% ethanol (1,000 mL) for 72 h. The solvent was removed using rotary evaporation at 40 °C, resulting in a semi-solid extract, which was then stored at 4 °C (Singha Ray et al., 2015).

### 2.4 Antibacterial testing

#### 2.4.1 Agar well diffusion

Bacterial suspensions standardized to a 0.5 McFarland turbidity were evenly applied to Mueller–Hinton agar plates. Wells, each measuring 6 mm in diameter, were filled with 100 μL of plant extracts at a concentration of 200 μg/mL. The experimental setup included a negative control of 1% DMSO and positive controls consisting of the antibiotics amoxicillin–clavulanic acid (30 μg) and tetracycline (30 μg). The plates were incubated at 37 °C for 24 h, after which the diameters of the inhibition zones were recorded in millimeters, with each measurement performed in triplicate (Bhattacharya and Chandra, 2014).

#### 2.4.2 Minimum inhibitory concentration (MIC)

Plant extract dilutions were prepared at concentrations of 50, 100, and 200 μg/mL. The MICs were determined through agar well inoculation against standardized bacterial strains (Klančnik et al., 2010; Cowan, 1999).

#### 2.4.3 SEM sample preparation of bacterial cells

The effect of the *M. peregrina* ethanol extract on the morphology of *Sh. sonnei* was investigated using SEM. Bacterial suspensions, at a concentration of 10^8^ CFU/mL, were treated with the *M. peregrina* extract to achieve a final concentration of 0.5 mg/L and were incubated overnight at 37 °C. A negative control consisting of untreated *Sh. sonnei* was processed in parallel. Post-incubation, the samples were washed with saline, centrifuged, and fixed with 2.5% glutaraldehyde at 4 °C for 2 h. The samples were then washed with phosphate buffer, post-fixed in 1% osmium tetroxide, dehydrated through a graded ethanol series, and subjected to critical point drying. Finally, The specimens were coated with an Au–Pd alloy and examined using a Quanta 250 scanning electron microscope (FEI, Hillsboro, OR, USA) at an accelerating voltage of 25 kV.

#### 2.4.4 Antibiotic interpretation

The inhibition zones were evaluated following the guidelines of the Clinical and Laboratory Standards Institute (CLSI, 2022) and the European Committee on Antimicrobial Susceptibility Testing (EUCAST, 2022). Bacteria were classified as susceptible (S), intermediate (I), or resistant (R) based on established reference breakpoints. Quality control strains were incorporated to ensure the validity, reproducibility, and accuracy of the assay. This standardization enabled meaningful comparisons between the antibacterial efficacy of the plant extracts and that of conventional antibiotics.

### 2.5 Phytochemical analysis

#### 2.5.1 Gas chromatography–mass spectrometry (GC–MS) analysis of phytochemical constituents

The ethanol extracts of *M. oleifera* and *M. peregrina* were subjected to GC–MS analysis to determine their chemical composition. This analysis utilized an Agilent 7890A GC system connected to a 5975C mass selective detector, equipped with a DB-5MS capillary column. Before injection, the extracts were filtered through a 22 μm membrane, and 1 μL of each sample was analyzed under standard conditions. Helium was used as the carrier gas, maintaining a constant flow rate of 1 mL/min. The injector temperature was set at 280 °C, while the column oven was programmed to reach a maximum of 300 °C. Mass spectra were recorded in electron impact (EI) mode with an ionization energy of 70 eV (Aljuhani et al., 2024).

#### 2.5.2 Qualitative phytochemical screening

To test for steroids, 1 gram of the plant extract was dissolved in a small volume of acetic acid, followed by the addition of a drop of concentrated H₂SO₄. The presence of steroids was confirmed by the appearance of a green tint (Talukdar and Chaudhary, 2010). For saponins, 2 g of the powdered sample was heated in 20 mL of distilled water. After filtration, 10 mL of the filtrate was combined with 5 mL of distilled water and shaken vigorously. The formation of froth indicated the presence of saponins (Alamzeb et al., 2013). The alkaloid test involved mixing 1 mL of Meyer’s reagent with 2 mL of the extract, and the appearance of a pale-yellow precipitate confirmed their presence (Talukdar and Chaudhary, 2010). For terpenoids, 0.2 g of each test sample was mixed with 2 mL of chloroform and 3 mL of concentrated H₂SO₄. The appearance of a red-brown color confirmed the presence of terpenoids (Alamzeb et al., 2013).

Flavonoid Detection: The Shinoda test was conducted by mixing 4 mL of the extract with 1.5 mL of a 50% methanol solution, followed by the addition of a small piece of magnesium. The mixture was then heated, and 5–6 drops of concentrated hydrochloric acid (HCl) were subsequently added. The appearance of a reddish hue indicated the presence of flavonoids (Talukdar and Chaudhary, 2010). Glycoside Detection: In the Molisch’s reagent test, 5 mL of Molisch’s reagent and concentrated H₂SO₄ were added to the extract. The development of a violet color confirmed the presence of glycosides (Alamzeb et al., 2013). The qualitative phytochemical analysis aimed to identify key bioactive compounds that may contribute to antibacterial activity, allowing for preliminary correlations with the observed inhibition zones. Although quantitative analyses, such as HPLC, can provide a more detailed assessment, this study prioritized qualitative screening as an initial approach. Future investigations should focus on quantifying these compounds to establish stronger correlations.

### 2.6 Statistical analysis

Data are presented as mean ± standard deviation (SD) from three replicates. Differences in means were deemed significant at a *p*-value of < 0.05, as determined by one-way ANOVA. GraphPad Prism 7.0 (GraphPad Software, Inc.) was utilized for data analysis and figure preparation.

## 3 Results

The study evaluated the antibacterial efficacy of leaf extracts from *M. oleifera* and *M. peregrina*, collected from the Wadi Ad-Dawasir region, against four Gram-negative bacterial strains. This investigation aimed to assess the inhibitory effects of ethanol, hot aqueous, and cold aqueous extracts, with a focus on identifying differences in antimicrobial activity specific to each species and solvent. Both *Moringa* species demonstrated significant antibacterial properties, with variation evident across the different extraction methods and bacterial strains tested.

### 3.1 Phytochemical analysis

**Figure 2:**
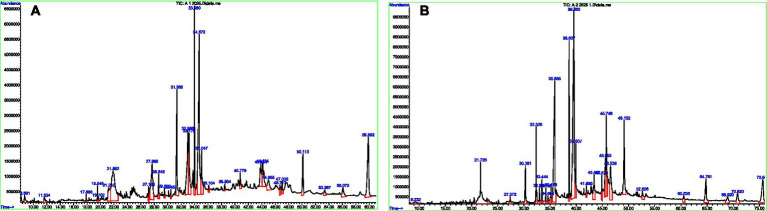
**(A)** GC–MS total ion chromatogram (TIC) of the *M. oleifera* leaf extract, showing the major phytochemical peaks across the retention time range. **(B)** GC–MS total ion chromatogram (TIC) of the *M. peregrina* leaf extract, illustrating the characteristic chemical profile and corresponding retention time peaks.

GC–MS analysis revealed the presence of 23 compounds in *M. oleifera* and 31 compounds in *M. peregrina*, highlighting their distinct phytochemical compositions. *M. oleifera* was characterized by a predominance of fatty acids, including cis-vaccenic acid (15.09%), stearic acid (13.85%), linoleic acid (9.16%), palmitic acid (8.55%), and oleic acid (5.38%), as well as terpenoids such as phytol (4.35%) and neophytadiene (3.06%). In contrast, *M. peregrina* was primarily composed of sterols, notably stigmasterol (18.52%) and *β*-sitosterol (16.05%), along with hydrocarbons and derivatives of long-chain alcohols (Figure 2; Table 1). The qualitative phytochemical assays supported these findings, identifying flavonoids, saponins, alkaloids, glycosides, and steroids in both plant extracts. Notably, saponins were more abundant in *M. peregrina*, whereas alkaloids and steroids were more prevalent in *M. oleifera*. It is important to note that terpenoids were not detected in either species, as illustrated in Table 2.

**Table 1:** Comparative GC–MS profile of the top 10 phytochemical constituents identified in *M. oleifera* and *M. peregrina* ethanol extracts.

Top 10 compounds in *M. oleifera*		Top 10 compounds in *M. peregrina*	
Compound	Area %	Compound	Area %
Cis-vaccenic acid	15.09%	Stigmasterol	18.52%
Stearic acid	13.85%	β-Sitosterol	16.05%
Linoleic acid	9.16%	Stearic acid	9.08%
Palmitic acid	8.55%	Hexacosane	6.17%
Oleic acid	5.38%	Heptacosanol	5.90%
Hentriacontane	5.10%	Linoleic acid derivatives	~6%
2(4H)-Benzofuranone derivative	6.99%	Hexatriacontane	5.46%
Neophytadiene	3.06%	Triacontane	2.33%
Phytol	4.35%	Dotriacontane	4.94%
Triacontane	4.84%	Hentriacontane	3.51%

**Table 2:** Phytochemical compounds in the leaf extracts *of M. oleifera* and *M. peregrina*.

Phytochemical compound	*M. oleifera*	*M. peregrina*
Glycosides	+	+
Steroids	++	+
Saponins	+	++
Alkaloids	++	+
Terpenoids	−	−
Flavonoids	+	+

### 3.2 Antibacterial activity

#### 3.2.1 Inhibition zone assay

**Figure 3:**
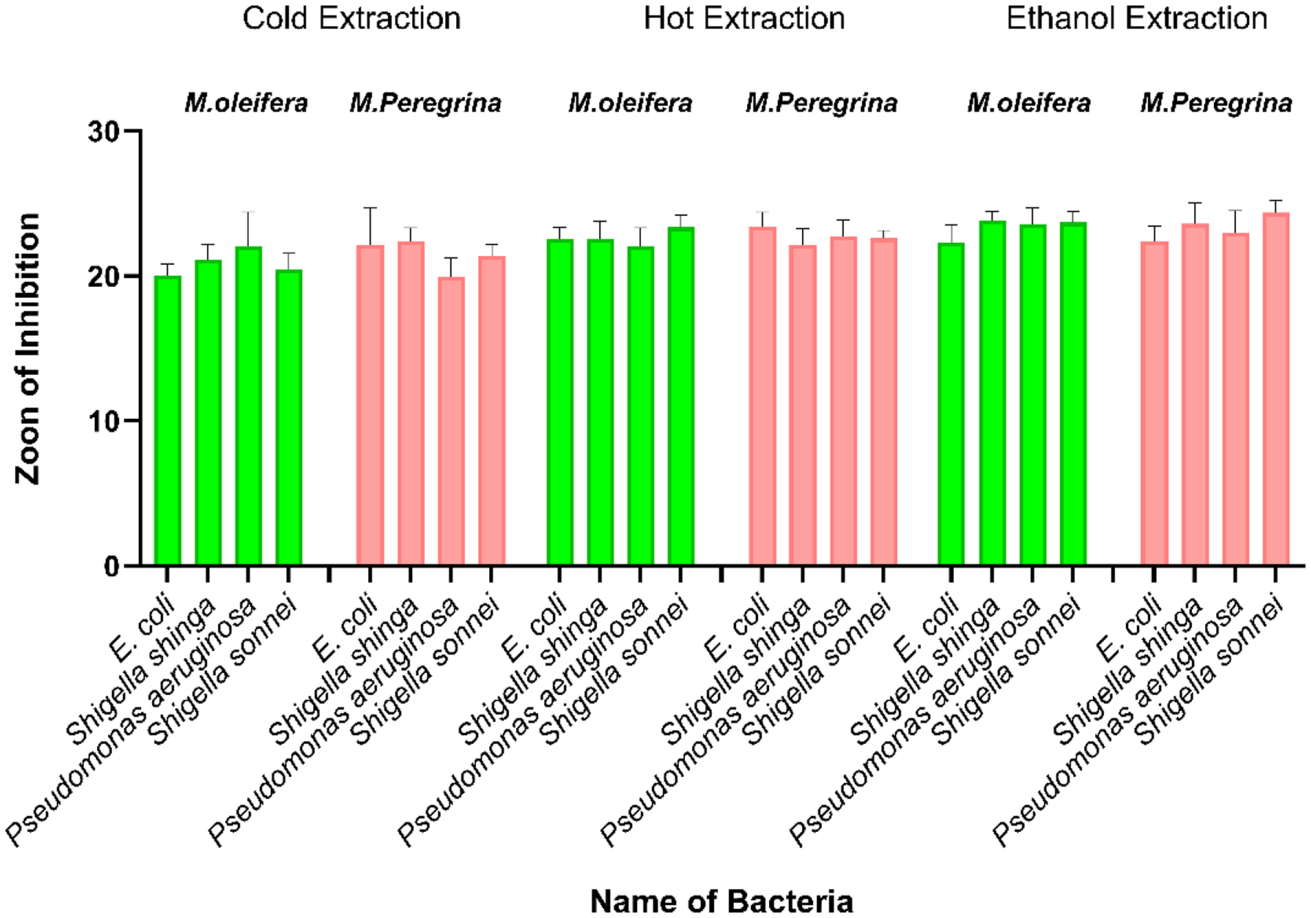
Antibacterial activity of *M. oleifera* and *M. peregrina* leaf extracts, showing the zone of inhibition against the tested organisms using cold aqueous, hot aqueous, and ethanol extracts (200 μg/mL). Values are presented as means ± standard deviations of triplicate determinations (*n* = 3). Statistically significant differences were considered at a *p*-value of < 0.05.

The study revealed that all extracts exhibited inhibitory effects against the tested Gram-negative bacteria, which included *Shigella shiga, Pseudomonas aeruginosa, Escherichia coli,* and *Shigella sonnei.* Among the extracts, ethanol extracts were the most effective, with the *M. peregrina* ethanol extract achieving an inhibition zone of 24.66 ± 0.17 mm against *Sh. sonnei*, surpassing the inhibition zone of 18.25 ± 1.69 mm observed with amoxicillin–clavulanic acid. The hot aqueous extracts demonstrated moderate antibacterial activity, with inhibition zones ranging from 21.77 ± 0.43 to 22.74 ± 0.39 mm for *M. oleifera* and from 22.69 ± 0.08 to 22.82 ± 1.07 mm for *M. peregrina*. Conversely, the cold aqueous extracts generally produced smaller inhibition zones, except in the case of *Shigella shiga*, where a more significant inhibitory effect was noted. Notably, none of the Gram-negative bacteria showed resistance to any of the extracts tested (Figure 3).

#### 3.2.2 Minimum inhibitory concentration (MIC)

The analysis of the MIC further corroborated the inhibitory effects of both species across various extraction solvents (Table 3). All extracts demonstrated significant antimicrobial activity, with MIC values consistently recorded at 50 μg/mL for *E. coli*, *Shigella shiga*, and *Sh. sonnei* in both *M. oleifera* and *M. peregrina*. A single deviation was observed: the cold aqueous extract of *M. oleifera* required 100 μg/mL to inhibit the growth of *P. aeruginosa*, whereas all other extracts—including those of *M. peregrina*—achieved inhibition at 50 μg/mL. This observation suggests that cold aqueous extraction may be less effective in solubilizing the active antibacterial constituents of *M. oleifera*, particularly against *P. aeruginosa*, a strain known for its high intrinsic resistance.

**Table 3:** Minimum inhibitory concentration (MIC) of plant extracts against the tested bacteria.

Bacteria species	*M. oleifera* (MIC W/V)			*M. peregrine* (MIC W/V)		
	Cold aqueous extract	Hot aqueous extract	Ethanol extract	Cold aqueous extract	Hot aqueous extract	Ethanol extract
*E. coli*	50	50	50	50	50	50
*Shigella shiga*	50	50	50	50	50	50
*Pseudomonas aeruginosa*	100	50	50	50	50	50
*Shigella sonnei*	50	50	50	50	50	50

#### 3.2.3 Comparative analysis

**Figure 4:**
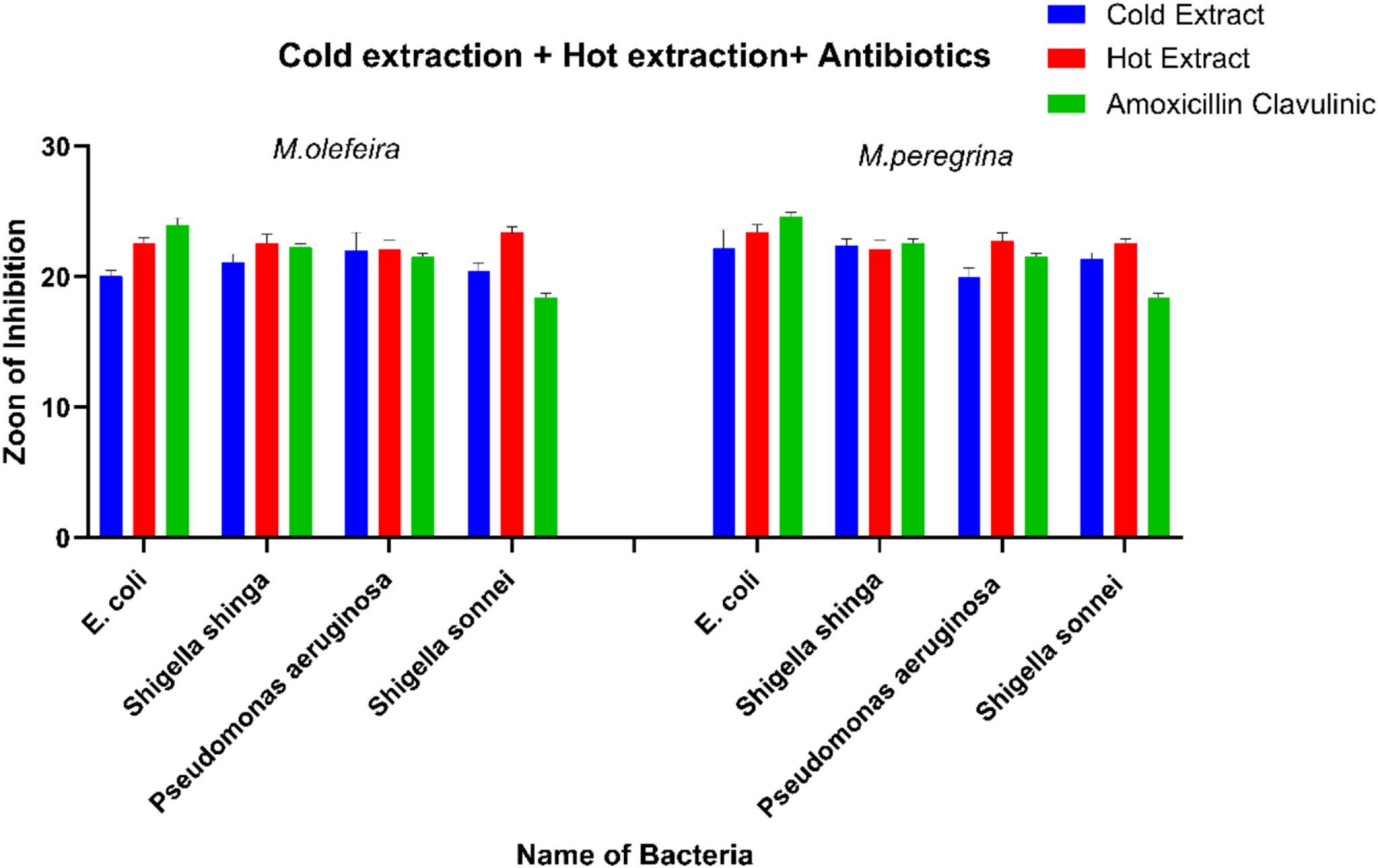
A comparison of the antibacterial activity of *M. oleifera* and *M. peregrina* leaf extracts using cold and hot extraction methods, alongside a standard antibiotic (amoxicillin–clavulanic acid). Bar charts represent the zones of inhibition (mm) against four bacterial strains (*E. coli*, *Sh. shiga*, *P. aeruginosa*, and *Sh. sonnei*). Blue bars indicate the cold extracts, red bars indicate the hot extracts, and green bars represent the antibiotic control. Error bars show the mean ± standard error for each treatmen**t.**

Table 3 presents the bactericidal activity of ethanol extracts from *M. oleifera* and *M. peregrina,* using amoxicillin–clavulanic acid as a control. *M. peregrina* exhibited the largest zones of inhibition, measuring 23.27 ± 0.52 mm against *P. aeruginosa* and 24.66 ± 0.17 mm against Sh. sonnei, indicating strong antibacterial activity against both Gram-negative bacteria. In comparison, *M. oleifera* exhibited slightly smaller inhibition zones of 22.32 ± 0.56 mm and 23.67 ± 0.25 mm, while amoxicillin–clavulanic acid demonstrated the smallest inhibition zones of 21.57 ± 1.25 mm and 18.25 ± 1.69 mm against the same bacteria. Notably, for *E. coli*, amoxicillin–clavulanic acid achieved the largest inhibition zone of 24.92 ± 1.26 mm, whereas *M. oleifera* showed the largest inhibition zone of 23.14 ± 0.16 mm against *Sh. shiga.* The ethanol extracts of *Moringa species* exhibited larger inhibition zones compared to the antibiotic (Figure 4). A comparison between the hot and cold extracts of *M. oleifera* and *M. peregrina,* with amoxicillin–clavulanic acid as a control, revealed that the antibiotic exerted a strong effect on *E. coli* and *Sh. shiga,* with inhibition zones of 24.92 ± 1.26 mm and 22.82 ± 1.41 mm, respectively. This was followed by the *M. peregrina* extracts, with an inhibition zone of 22.69 ± 0.22 mm for *E. coli,* and the *M. oleifera* extracts, with an inhibition zone of 22.58 ± 0.27 mm for *Sh. shiga*. However, the *M. peregrina* extracts exhibited the highest inhibition zones of 22.69 ± 0.08 mm and 22.82 ± 1.07 mm against *P. aeruginosa* and *Sh. sonnei*, respectively, followed by the *M. oleifera* extracts, with inhibition zones of 21.77 ± 0.43 mm and 22.74 ± 0.39 mm, respectively. Amoxicillin–clavulanic acid showed the lowest inhibition zones for these two bacteria, measuring 21.57 ± 1.25 mm and 18.25 ± 1.69 mm. It is noteworthy that amoxicillin–clavulanic acid, used as a positive control, exhibited the least effect on *Sh. sonnei*, with an inhibition zone of 18.25 ± 1.69 mm. The antimicrobial effects of the cold extracts of *M. oleifera* and *M. peregrina* were compared with those of amoxicillin–clavulanic acid. Amoxicillin–clavulanic acid demonstrated the highest inhibition zones of 24.92 ± 1.26 mm, 22.82 ± 1.41 mm, and 21.57 ± 1.25 mm against *E. coli*, *Sh. shiga*, and *P. aeruginosa,* respectively—the Gram-negative bacteria evaluated in this study. However, it exhibited a smaller inhibition zone of 18.25 ± 1.69 mm against *Sh. sonnei*. The inhibition zones of *M. oleifera* cold aqueous extracts were higher for *E. coli* and *Pseudomonas aeruginosa* compared to those of *M. peregrina* extracts. Conversely, the *M. peregrina* extracts outperformed in terms of inhibition zones against *Sh. shiga* and *Sh. sonnei*, measuring 21.69 ± 0.08 mm and 20.61 ± 0.39 mm, respectively. It was observed that amoxicillin–clavulanic acid, with an inhibition zone of 18.25 ± 1.69 mm, was less effective than the cold extracts of the *Moringa species* against *Sh. sonnei*.

### 3.3 Morphological changes in *Sh. sonnei* treated with the *Moringa peregrina* extract

**Figure 5:**
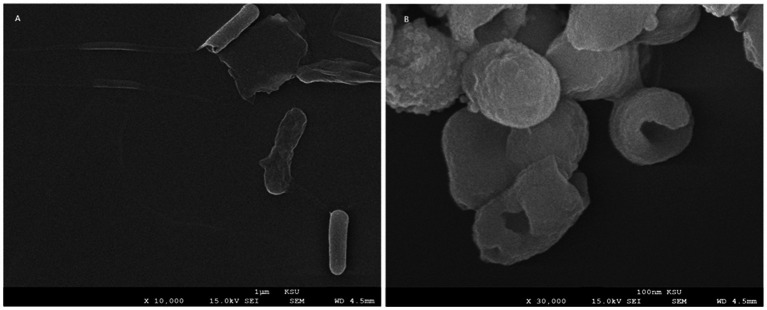
Scanning electron microscopy (SEM) of *S. sonnei* before and after treatment with the ethanol extract of *M. peregrina*. **(A)** Untreated *Sh. sonnei* cells are characterized by their smooth, elongated rod-shaped morphology, with intact outer membrane surfaces that exhibit uniform contours. There was no indication of membrane disruption, perforation, or cytoplasmic leakage, suggesting that their structural integrity was preserved. **(B)** In contrast, *Sh. sonnei* cells treated with the *M. peregrina* ethanol extract (0.5 mg/mL) showed marked ultrastructural damage. This included surface collapse, deep membrane perforation, irregular cavities, wall rupture, and leakage of cytoplasmic contents. Numerous cells appeared distorted, shrunken, and structurally fragmented, providing evidence of the extract’s bactericidal effect on membrane structure.

The untreated *Sh. sonnei* cells retained their typical smooth, elongated rod-shaped morphology, characteristic of healthy Gram-negative bacilli. These cells exhibited intact and well-defined surfaces and a consistent cylindrical structure, with no signs of membrane disruption or cytoplasmic leakage, indicating that their structural integrity was preserved (Figure 5A). In stark contrast, cells treated with the *M. peregrina* extract displayed significant morphological changes, indicative of the extract’s potent antibacterial effects. The treated cells experienced severe cell wall disruption, including surface collapse, wrinkling, and disintegration of the outer membrane. This was further evidenced by the presence of deep pits, perforations, and irregular cavities, suggesting a loss of membrane integrity. Many cells exhibited cytoplasmic leakage and lytic features, appearing shrunken, distorted, or fragmented, with some collapsing into amorphous forms. Collectively, these observations underscore the extract’s ability to compromise bacterial membrane integrity and induce cell death (Figure 5B).

## 4 Discussion

The therapeutic potential of *M. oleifera* is widely acknowledged, largely due to its rich array of phytochemicals, including phenolic acids, flavonoids, and alkaloids, which are known to compromise bacterial membranes, inhibit cell wall synthesis, and disrupt microbial metabolism. This study revealed that leaf extracts from both *M. oleifera* and *M. peregrina* exhibited antibacterial properties against Gram-negative bacteria, despite the inherent resistance conferred by their double-membrane cell envelope. The extracts prepared using hot water, cold water, and ethanol effectively inhibited the growth of *E. coli*, *Sh. shiga*, *P. aeruginosa*, and *Sh. sonnei*, supporting previous findings that *Moringa* extracts are potent natural antimicrobial agents (Klančnik et al., 2010; Cowan, 1999). Consistent with earlier research (Cowan, 1999; Kowalska-Krochmal and Dudek-Wicher, 2021), the ethanol extracts produced the most significant inhibition zones, indicative of the superior solubility and extraction efficiency of bioactive compounds in organic solvents. Our MIC results further confirmed the strong antibacterial activity of both *Moringa species*, with the majority of extracts inhibiting bacterial growth at a concentration of 50 μg/mL. The only exception was the cold aqueous extract of *M. oleifera* against *P. aeruginosa*, which required a higher MIC of 100 μg/mL. This finding is consistent with the well-documented resilience of *P. aeruginosa* due to its restrictive outer membrane and efflux systems (Hancock and Speert, 2000).

The superior performance of hot water extracts over cold water extracts aligns with previous research indicating that extraction temperature plays a crucial role in determining phytochemical concentration and efficacy (Cowan, 1999). These findings collectively affirm the therapeutic potential of the *Moringa species*, consistent with earlier studies demonstrating their antibacterial properties (Rahman et al., 2009). The GC–MS analysis provided further insights into the phytochemical variation underlying these antibacterial effects. *M. oleifera* was found to be rich in unsaturated fatty acids—such as cis-vaccenic, linoleic, and oleic acids—recognized for their potent anti-inflammatory and antioxidant activities (Anwar and Rashid, 2007; Leone et al., 2015). Additionally, terpenoids such as phytol and neophytadiene, known for their antimicrobial and cytotoxic properties, likely contributed to the extract’s activity. Conversely, *M. peregrina* exhibited a sterol-dominated profile, with stigmasterol and *β*-sitosterol constituting more than one-third of the extract. These phytosterols are associated with cholesterol-lowering, anticancer, and immunomodulatory effects (Kumar et al., 2019). The phytochemical distinction—fatty-acid dominance in *M. oleifera* and sterol enrichment in *M. peregrina*—aligns with previous reports on species-specific chemical variation within the genus *Moringa* (Al-Owaisi et al., 2014). Notably, similar sterol-driven antimicrobial actions were documented in a previous study (Xu et al., 2025), where sterol-like compounds induced membrane disruption in Gram-negative pathogens, supporting our observed potency of sterol-rich *M. peregrina*. SEM imaging further substantiated the antibacterial mechanisms inferred from GC–MS data. Untreated *Sh. sonnei* cells maintained smooth, intact rod-shaped morphology, whereas cells exposed to the *M. peregrina* extract exhibited severe structural damage, including membrane collapse, perforation, and leakage of intracellular contents—which are the characteristics of bactericidal action. These morphological changes strongly suggest that phytosterols (stigmasterol and *β*-sitosterol) and fatty acids (linoleic derivatives), as identified by GC–MS, compromise membrane integrity and induce cell death. Comparable membrane-disruptive effects of plant-derived sterols have also been reported in Journal of Medicinal Chemistry, 2025 (Xu et al., 2025), providing external validation for our observations. Overall, the combined antimicrobial assays, GC–MS profiling, and SEM imaging indicated that both *M. oleifera* and *M. peregrina* possess significant antibacterial potential, with *M. peregrina* demonstrating comparatively stronger activity—likely due to its sterol-rich chemical composition. These results underscore the pharmacological value of the *Moringa species* native to the Wadi Ad-Dawasir region and highlight the importance of preserving local biodiversity. Future research should focus on isolating individual bioactive compounds, evaluating synergistic interactions, and validating the efficacy through *in vivo* studies. These investigations may ultimately support the development of *Moringa*-derived therapeutic agents capable of combating Gram-negative bacterial infections, particularly in the context of rising antibiotic resistance.

## 5 Conclusion

This research establishes that leaf extracts from *M. oleifera* and *M. peregrina*, collected from the Wadi Ad-Dawasir region, possess notable antibacterial activity against clinically significant Gram-negative bacteria. The ethanol extracts were particularly effective, with *M. peregrina* demonstrating greater efficacy than amoxicillin–clavulanic acid against *Sh. sonnei* (24.66 ± 0.17 mm vs. 18.25 ± 1.69 mm) and showing superior overall antibacterial activity. GC–MS analysis revealed that *M. peregrina* is rich in sterols, while *M. oleifera* contains a higher concentration of fatty acids, suggesting that these phytochemicals contribute to their antibacterial effectiveness. The chemical profiles corresponded with the cellular effects observed, as SEM imaging showed direct membrane-targeting actions, including significant surface collapse, perforation, cytoplasmic leakage, and severe structural deformation, in *Sh. sonnei* cells treated with the extracts. These ultrastructural changes confirm the bactericidal action of the extracts, supporting a mechanism of membrane destabilization rather than simple growth inhibition. The minimum inhibitory concentration (MIC) values, predominantly 50 μg/mL, further confirm the strong antimicrobial activity of both species at low concentrations, highlighting their therapeutic potential. Overall, these results emphasize the potential of *Moringa species*, especially *M. peregrina*, as promising phytotherapeutic agents against drug-resistant Gram-negative infections. Future investigations should focus on isolating bioactive compounds, assessing synergistic effects with existing antibiotics, and conducting *in vivo* studies to advance their clinical applications.

## Data Availability

The original contributions presented in the study are included in the article/supplementary material, further inquiries can be directed to the corresponding author.

## References

[ref1] Abe R. Ohtani K. (2013). An ethnobotanical study of medicinal plants and traditional therapies on Batan Island, the Philippines. J. Ethnopharmacol. 145, 554–565. doi: 10.1016/j.jep.2012.11.029, 23183086

[ref2] Alamzeb M. Khan M. R. Ali S. Shah S. Q. (2013). Antimicrobial properties of extracts and compounds isolated from *Berberis jaeschkeana*. Bangladesh J. Pharmacol. 8, 107–109. doi: 10.3329/bjp.v8i2.13551

[ref3] Al-Dabbas M. M. Ahmad R. Ajo R. Y. Abulaila K. Akash M. Al-Ismail K. (2010). Chemical composition and oil components in seeds of *Moringa peregrina* (Forssk) Fiori. Crop. Res. 40:2.

[ref4] Aljuhani S. Rizwana H. Aloufi A. S. Alkahtani S. Albasher G. Almasoud H. . (2024). Antifungal activity of *Carica papaya* fruit extract against Microsporum canis: *in vitro* and *in vivo* study. Front. Microbiol. 15:1399671. doi: 10.3389/fmicb.2024.1399671, 38803379 PMC11128596

[ref5] Al-Owaisi M. Al-Hadiwi N. Khan S. A. (2014). GC-MS analysis, determination of total phenolics, flavonoid content and free radical scavenging activities of various crude extracts of *Moringa peregrina* (Forssk.) Fiori leaves. Asian Pac. J. Trop. Biomed. 4, 964–970. doi: 10.12980/APJTB.4.201414B295

[ref6] Andersson D. I. (2003). Persistence of antibiotic resistant bacteria. Curr. Opin. Microbiol. 6, 452–456. doi: 10.1016/j.mib.2003.09.001, 14572536

[ref7] Anwar F. Rashid U. (2007). Physico-chemical characteristics of *Moringa oleifera* seeds and seed oil from a wild provenance of Pakistan. Pak. J. Bot. 39, 1443–1453. doi: 10.5555/20083055010

[ref8] Avilés-Gaxiola S. León-Félix J. Jiménez-Nevárez Y. B. Angulo-Escalante M. A. Ramos-Payán R. Colado-Velázquez J. III . (2021). Antioxidant and anti-inflammatory properties of novel peptides from *Moringa oleifera* lam. leaves. S. Afr. J. Bot. 141, 466–473. doi: 10.1016/j.sajb.2021.05.033

[ref9] Banerji R. Verma S. Pushpangadan P. (2003). Oil potential of Moringa.

[ref10] Benmohamed M. Guenane H. Messaoudi M. Zahnit W. Egbuna C. Sharifi-Rad M. . (2023). Mineral profile, antioxidant, anti-inflammatory, antibacterial, anti-urease and anti-α-amylase activities of the unripe fruit extracts of *Pistacia atlantica*. Molecules 28:349. doi: 10.3390/molecules28010349, 36615545 PMC9824078

[ref11] Bhattacharya K. Chandra G. (2014). Phagodeterrence, larvicidal and oviposition deterrence activity of *Tragia involucrata* L. (Euphorbiaceae) root extractives against vector of lymphatic filariasis *Culex quinquefasciatus* (Diptera: Culicidae). Asian Pac. J. Trop. Dis. 4, S226–S232. doi: 10.1016/S2222-1808(14)60444-8

[ref12] Boulos L. Geraniaceae-Boraginaceae. (No Title). (2000)

[ref13] Brilhante R. S. N. Sales J. A. Pereira V. S. Castelo-Branco D. d. S. C. M. de Aguiar Coriro R. de Souza Sampaio C. M. . (2017). Research advances on the multiple uses of *Moringa oleifera*: a sustainable alternative for socially neglected population. Asian Pac. J. Trop. Med. 10, 621–630. doi: 10.1016/j.apjtm.2017.07.002 28870337

[ref14] Bugno A. Nicoletti M. A. Almodóvar A. A. Pereira T. C. Auricchio M. T. (2007). Antimicrobial efficacy of *Curcuma zedoaria* extract as assessed by linear regression compared with commercial mouthrinses. Braz. J. Microbiol. 38, 440–445. doi: 10.1590/S1517-83822007000300011

[ref15] Cao J. Shi T. Wang H. Zhu F. Wang J. Wang Y. . (2023). *Moringa oleifera* leaf protein: extraction, characteristics and applications. J. Food Compos. Anal. 119:105234. doi: 10.1016/j.jfca.2023.105234

[ref16] Cowan M. M. (1999). Plant products as antimicrobial agents. Clin. Microbiol. Rev. 12, 564–582. doi: 10.1128/CMR.12.4.564 10515903 PMC88925

[ref17] Dilhuydy J.-M. (2003). Patients’ propensity for complementary and alternative medicine (CAM): a reality which physicians can neither ignore nor deny. Bull. Cancer 90, 623–628. 12957804

[ref18] Doughari J. Elmahmood A. Manzara S. (2007). Studies on the antibacterial activity of root extracts of *Carica papaya* L. Afr. J. Microbiol. Res. 1, 37–41.

[ref19] El-Hak H. N. G. Moustafa A. R. A. Mansour S. R. (2018). Toxic effect of Moringa peregrina seeds on histological and biochemical analyses of adult male albino rats. Toxicol. Rep. 5, 38–45. doi: 10.1016/j.toxrep.2017.12.012, 29276689 PMC5730415

[ref20] Fahey J. W. (2005). *Moringa oleifera*: a review of the medical evidence for its nutritional, therapeutic, and prophylactic properties. Part 1. Trees Life J. 1, 1–15.

[ref21] Fahey J. W. Olson M. E. Stephenson K. K. Wade K. L. Chodur G. M. Odee D. . (2018). The diversity of chemoprotective glucosinolates in Moringaceae (*Moringa* spp.). Sci. Rep. 8:7994. doi: 10.1038/s41598-018-26058-4, 29789618 PMC5964242

[ref22] Hancock R. E. Speert D. P. (2000). Antibiotic resistance in *Pseudomonas aeruginosa*: mechanisms and impact on treatment. Drug Resist. Updat. 3, 247–255. doi: 10.1054/drup.2000.0152, 11498392

[ref23] Hernandez-Aguilar C. Dominguez-Pacheco A. Valderrama-Bravo C. Cruz-Orea A. Ortiz E. M. Ivanov R. . (2021). Photoacoustic characterization of wheat bread mixed with *Moringa oleifera*. Curr. Res. Food Sci. 4, 521–531. doi: 10.1016/j.crfs.2021.07.008, 34401748 PMC8350460

[ref24] Klančnik A. Piskernik S. Jeršek B. Možina S. S. (2010). Evaluation of diffusion and dilution methods to determine the antibacterial activity of plant extracts. J. Microbiol. Methods 81, 121–126. doi: 10.1016/j.mimet.2010.02.004 20171250

[ref25] Kowalska-Krochmal B. Dudek-Wicher R. (2021). The minimum inhibitory concentration of antibiotics: methods, interpretation, clinical relevance. Pathogens 10:165. doi: 10.3390/pathogens10020165, 33557078 PMC7913839

[ref26] Kumar G. Gupta R. Sharan S. Roy P. Pandey D. M. (2019). Anticancer activity of plant leaves extract collected from a tribal region of India. 3 Biotech 9:399. doi: 10.1007/s13205-019-1927-x, 31656737 PMC6790204

[ref27] Leone A. Spada A. Battezzati A. Schiraldi A. Aristil J. Bertoli S. (2015). Cultivation, genetic, ethnopharmacology, phytochemistry and pharmacology of *Moringa oleifera* leaves: an overview. Int. J. Mol. Sci. 16, 12791–12835. doi: 10.3390/ijms160612791, 26057747 PMC4490473

[ref28] Manyi-Loh C. Mamphweli S. Meyer E. Okoh A. (2018). Antibiotic use in agriculture and its consequential resistance in environmental sources: potential public health implications. Molecules 23:795. doi: 10.3390/molecules23040795, 29601469 PMC6017557

[ref29] Medina E. Pieper D. Stadler M. Dersch P. (2016). How to overcome the antibiotic crisis: Facts, challenges, technologies and future perspectives. Cham, Switzerland: Springer.

[ref30] Morton J. F. (1991). The horseradish tree, *Moringa pterygosperma* (Moringaceae)—a boon to arid lands? Econ. Bot. 45, 318–333. doi: 10.1007/BF02887070

[ref31] Murray C. J. Ikuta K. S. Sharara F. Swetschinski L. Aguilar G. R. Gray A. . (2022). Global burden of bacterial antimicrobial resistance in 2019: a systematic analysis. Lancet 399, 629–655. doi: 10.1016/S0140-6736(21)02724-0 35065702 PMC8841637

[ref32] Pandey A. Pandey R. Tripathi P. Gupta P. Haider J. Bhatt S. . (2012). *Moringa oleifera* lam. (Sahijan)-a plant with a plethora of diverse therapeutic benefits: an updated retrospection medicinal and aromatic plants. Medicinal Aromatic Plants 1, 1–8. doi: 10.4172/map.1000101

[ref33] Peter K. V. (1979). English, Journal article, 0019-4875, 23, (4), Indian Horticulture, (17–18), Drumstick, a multi-purpose vegetable.

[ref34] Qixia G. Zijun S. Shihuan T. Zhiyong L. (2020). Research progress of chemical constituents and pharmacological effects of *Moringa Oleifera*. Herald Med. 39, 350–359. doi: 10.3870/j.issn.1004-0781.2020.03.01

[ref35] Quattrocchi U. (2012). CRC world dictionary of medicinal and poisonous plants: Common names, scientific names, eponyms, synonyms, and etymology (5 volume set). Boca Raton, FL: CRC press.

[ref36] Rahman M. M. Sheikh M. M. I. Sharmin S. A. Islam M. S. Rahman M. A. Rahman M. M. . (2009). Antibacterial activity of leaf juice and extracts of *Moringa oleifera* lam. Against some human pathogenic bacteria. CMU J Nat Sci. 8:219.

[ref37] Rhen M. Eriksson S. Clements M. Bergström S. Normark S. J. (2003). The basis of persistent bacterial infections. Trends Microbiol. 11, 80–86. doi: 10.1016/S0966-842X(02)00038-0, 12598130

[ref38] Sengupta A. Gupta M. (1970). Studies on the seed fat composition of Moringaceae family. Fette Seifen Anstrichmittel 72, 6–10. doi: 10.1002/lipi.19700720103

[ref39] Sharif M. Banik G. R. (2006). Status and utilization of medicinal plants in Rangamati of Bangladesh. Res. J. Agric. Biol. Sci. 2, 268–273.

[ref40] Sharifi Rad J. Hoseini Alfatemi S. M. Sharifi Rad M. Iriti M. (2014). Free radical scavenging and antioxidant activities of different parts of *Nitraria schoberi* L. J. Biol. Act. Prod. Nat. 4, 44–51. doi: 10.1080/22311866.2014.890070

[ref41] Sharifi-Rad M. Mohanta Y. K. Pohl P. Jaradat N. Aboul-Soud M. A. Zengin G. (2022). Variation of phytochemical constituents, antioxidant, antibacterial, antifungal, and anti-inflammatory properties of *Grantia aucheri* (Boiss.) at different growth stages. Microb. Pathog. 172:105805. doi: 10.1016/j.micpath.2022.105805 36179974

[ref42] Sharifi-Rad M. Panda J. Mohanta Y. K. Pohl P. Zengin G. Moloney M. G. (2025). Essential oil of *Cleome coluteoides* (Boiss.): phytochemical constituents, antioxidant, antimicrobial, antiproliferative, anti-inflammatory, enzymatic inhibition, and xanthine oxidase inhibitory properties. J. Herb. Med. 52:101036. doi: 10.1016/j.hermed.2025.101036

[ref43] Sharma V. Paliwal R. Sharma P. Sharma S. (2011). Phytochemical analysis and evaluation of antioxidant activities of hydro-ethanolic extract of *Moringa oleifera* lam. Pods. J. Pharm. Res. 4, 554–557. doi: 10.5555/20113124678

[ref44] Singha Ray A. Bhattacharya K. Chandra G. (2015). Target specific larvicidal effect of *Capparis zeylanica* (Capparaceae) foliages against filarial vector *Culex quinquefasciatus* say (1823). Int J Pharm. Bio. Sci 6, 139–148.

[ref45] Talukdar A. Chaudhary B. (2010). Phytochemical screening of ethanolic extracts of *Rubiacordiofolia*. Pharma Bio Sci. 1, 530–536.

[ref46] Trigo C. Castello M. L. Ortola M. D. Garcia-Mares F. J. Desamparados Soriano M. (2020). *Moringa oleifera*: an unknown crop in developed countries with great potential for industry and adapted to climate change. Foods 10:31. doi: 10.3390/foods10010031, 33374455 PMC7824577

[ref47] Velez R. Sloand E. (2016). Combating antibiotic resistance, mitigating future threats and ongoing initiatives. J. Clin. Nurs. 25, 1886–1889. doi: 10.1111/jocn.13246, 26991135

[ref48] Vijaya K. Ananthan S. (1997). Microbiological screening of Indian medicinal plants with special reference to enteropathogens. J. Altern. Complement. Med. 3, 13–20. doi: 10.1089/acm.1997.3.13, 9395690

[ref49] Willis K. (2017). State of the world's plants 2017. Richmond: Royal Botanics Gardens Kew. 29144713

[ref50] Xu Y. Chen G. Muema F. W. Xiao J. Guo M. (2024). Most recent research progress in *Moringa oleifera*: bioactive phytochemicals and their correlated health promoting effects. Food Rev. Int. 40, 740–770. doi: 10.1080/87559129.2023.2195189

[ref51] Xu T. Xue Z. Li X. Zhang M. Yang R. Qin S. . (2025). Development of membrane-targeting Osthole derivatives containing Pyridinium quaternary ammonium moieties with potent anti-methicillin-resistant *Staphylococcus aureus* properties. J. Med. Chem. 68, 7459–7475. doi: 10.1021/acs.jmedchem.4c03167, 40205941

[ref52] Ziebuhr W. Ohlsen K. Karch H. Korhonen T. Hacker J. (1999). Evolution of bacterial pathogenesis. Cell Mol Life Sci 56, 719–728. doi: 10.1007/s000180050018, 11212331 PMC11147097

